# High temporal and spatial diversity in marine RNA viruses implies that they have an important role in mortality and structuring plankton communities

**DOI:** 10.3389/fmicb.2014.00703

**Published:** 2014-12-15

**Authors:** Julia A. Gustavsen, Danielle M. Winget, Xi Tian, Curtis A. Suttle

**Affiliations:** ^1^Department of Earth, Ocean and Atmospheric Sciences, University of British ColumbiaVancouver, BC, Canada; ^2^Bioinformatics Graduate Program, Faculty of Science, University of British ColumbiaVancouver, BC, Canada; ^3^Departments of Botany, and Microbiology & Immunology, University of British ColumbiaVancouver, BC, Canada; ^4^Canadian Institute for Advanced ResearchToronto, ON, Canada

**Keywords:** RNA viruses, phytoplankton mortality, viral ecology, pyrosequencing, viral diversity, *Picornavirales*

## Abstract

Viruses in the order *Picornavirales* infect eukaryotes, and are widely distributed in coastal waters. Amplicon deep-sequencing of the RNA dependent RNA polymerase (RdRp) revealed diverse and highly uneven communities of picorna-like viruses in the coastal waters of British Columbia (BC), Canada. Almost 300 000 pyrosequence reads revealed 145 operational taxonomic units (OTUs) based on 95% sequence similarity at the amino-acid level. Each sample had between 24 and 71 OTUs and there was little overlap among samples. Phylogenetic analysis revealed that some clades of OTUs were only found at one site; whereas, other clades included OTUs from all sites. Since most of these OTUs are likely from viruses that infect eukaryotic phytoplankton, and viral isolates infecting phytoplankton are strain-specific; each OTU probably arose from the lysis of a specific phytoplankton taxon. Moreover, the patchiness in OTU distribution, and the high turnover of viruses in the mixed layer, implies continuous infection and lysis by RNA viruses of a diverse array of eukaryotic phytoplankton taxa. Hence, these viruses are likely important elements structuring the phytoplankton community, and play a significant role in nutrient cycling and energy transfer.

## Introduction

Viruses are highly abundant and widespread in the oceans (Bergh et al., 1989; Suttle, 2005). Beyond their impacts on host mortality, viruses are significant mediators of biogeochemical processes, horizontal gene transfer, and host community diversity in the oceans (Fuhrman, 1999; Wilhelm and Suttle, 1999; Suttle, 2005). Marine viruses are important pathogens of phytoplankton (Brussaard, 2004), and have been implicated in the termination of blooms (Nagasaki et al., 1994; Schroeder et al., 2003) and with succession in phytoplankton communities (Mühling et al., 2005). Viruses have been characterized that infect a wide variety of phytoplankton such as haptophytes (Bratbak et al., 1993), prasinophytes (Mayer and Taylor, 1979; Brussaard, 2004; Brussaard et al., 2004; Derelle et al., 2008), chlorophytes (Van Etten et al., 1981), diatoms (Shirai et al., 2008), and dinoflagellates (Tomaru et al., 2004). Viruses infecting eukaryotic phytoplankton generally have very narrow host ranges (Short, 2012).

Viruses infecting marine phytoplankton have genomes comprised of double-stranded DNA (dsDNA), single-stranded DNA (ssDNA), double-stranded RNA (dsRNA), and single-stranded RNA (ssRNA) (as reviewed in Short, 2012). Their genomes and particle sizes range from very large dsDNA viruses in the *Phycodnaviridae* to very small ssDNA and ssRNA viruses belonging to the genus *Bacilladnavirus* and order *Picornavirales*, respectively. The order *Picornavirales* is comprised of positive-sense, ssRNA viruses that infect eukaryotes (Le Gall et al., 2008), including ecologically important marine protists. These viruses are small (25–35 nm), icosahedral, and have a conserved genomic organization that includes a replication area comprised of a type III helicase, a 3C-like proteinase, and a type I RNA dependent RNA polymerase (Sanfaçon et al., 2009). Isolates in the *Picornavirales* that are pathogens of marine protists infect a wide diversity of hosts including the bloom-forming raphidophyte *Heterosigma akashiwo* (Tai et al., 2003) (viral family *Marnaviridae*), the thraustochytrid *Aurantiochytrium s*p. (Takao et al., 2006; Yokoyama and Honda, 2007) (proposed viral genus *Labyrnavirus*) and the cosmopolitan diatoms *Rhizosolenia setigera* (Nagasaki et al., 2004) and *Chaetoceros socialis* (Tomaru et al., 2009) (proposed viral genus *Bacillarnavirus*). Viruses in the *Picornavirales* appear to be common and widely distributed in coastal waters (Culley et al., 2003; Culley and Steward, 2007).

Metagenomic and targeted gene studies are uncovering the diversity of marine RNA viruses. For example, phylogenetic analysis of RNA-dependent RNA polymerase (RdRp) sequences from seawater samples supports a monophyletic marine group within the *Picornavirales* (Culley et al., 2003, 2014; Culley and Steward, 2007; Tomaru et al., 2009) and several divergent clades within this marine group (Culley et al., 2003, 2014; Culley and Steward, 2007). Additionally, metagenomic analyses reveal that there are numerous sequences from aquatic RNA viruses that cannot be assigned to known taxa (Culley et al., 2006, 2014; Djikeng et al., 2009; Steward et al., 2012). Despite the high diversity of marine RNA viruses (Lang et al., 2009), the spatial and temporal distribution of different phylogenetic groups remains unreported, although there is evidence that the taxonomic structure of marine RNA viral communities is highly uneven. For example, in one sample from a metagenomic study from the coastal waters of British Columbia, 59% of the reads assembled into a single contig, while in a second sample 66% of the reads fell into four contigs, with most falling into two genotypes (Culley et al., 2006). However, with only a few 100 reads in total from the two samples, the coverage of the communities was low. Similarly, RNA viral metagenomic data from a freshwater lake (Djikeng et al., 2009) showed little identical sequence overlap among communities, although there was broad taxonomic similarity over time within a location.

Ecological questions about the distribution of marine viruses over time and space have been examined more extensively in bacteriophages, particularly those infecting cyanobacteria. For example, some data reveal no clear patterns of biogeography in cyanophage isolates locally (Clasen et al., 2013), regionally (Jameson et al., 2011) or more globally (Huang et al., 2010). Other data have shown patterns at a regional scale (Marston et al., 2013), although communities in basins that were connected were most similar and those that were separated by land or current boundaries were the least similar. Other data for marine bacteriophages have shown temporal variability (Chen et al., 2009; Wang et al., 2011; Chow and Fuhrman, 2012; Clasen et al., 2013; Marston et al., 2013). If the dynamics of marine bacteriophages and marine RNA viruses are similar, some RNA viral taxa will persist temporally and spatially, while other taxa will be detected sporadically. To test this hypothesis, we examined two samples, taken 5 months apart at the same location, and three samples taken within hours of each other, but 20 km apart in the same coastal basin.

We used high-throughput 454 pyrosequencing to obtain deep coverage of RdRp amplicon sequences and compare the richness of viruses in the *Picornavirales* among samples from the coastal waters of British Columbia, Canada. The results revealed a phylogenetically diverse and spatially variable community of viruses, suggesting that taxon-specific lytic events are important in shaping the phytoplankton community.

## Materials and methods

### Sampling locations

**Figure 1:**
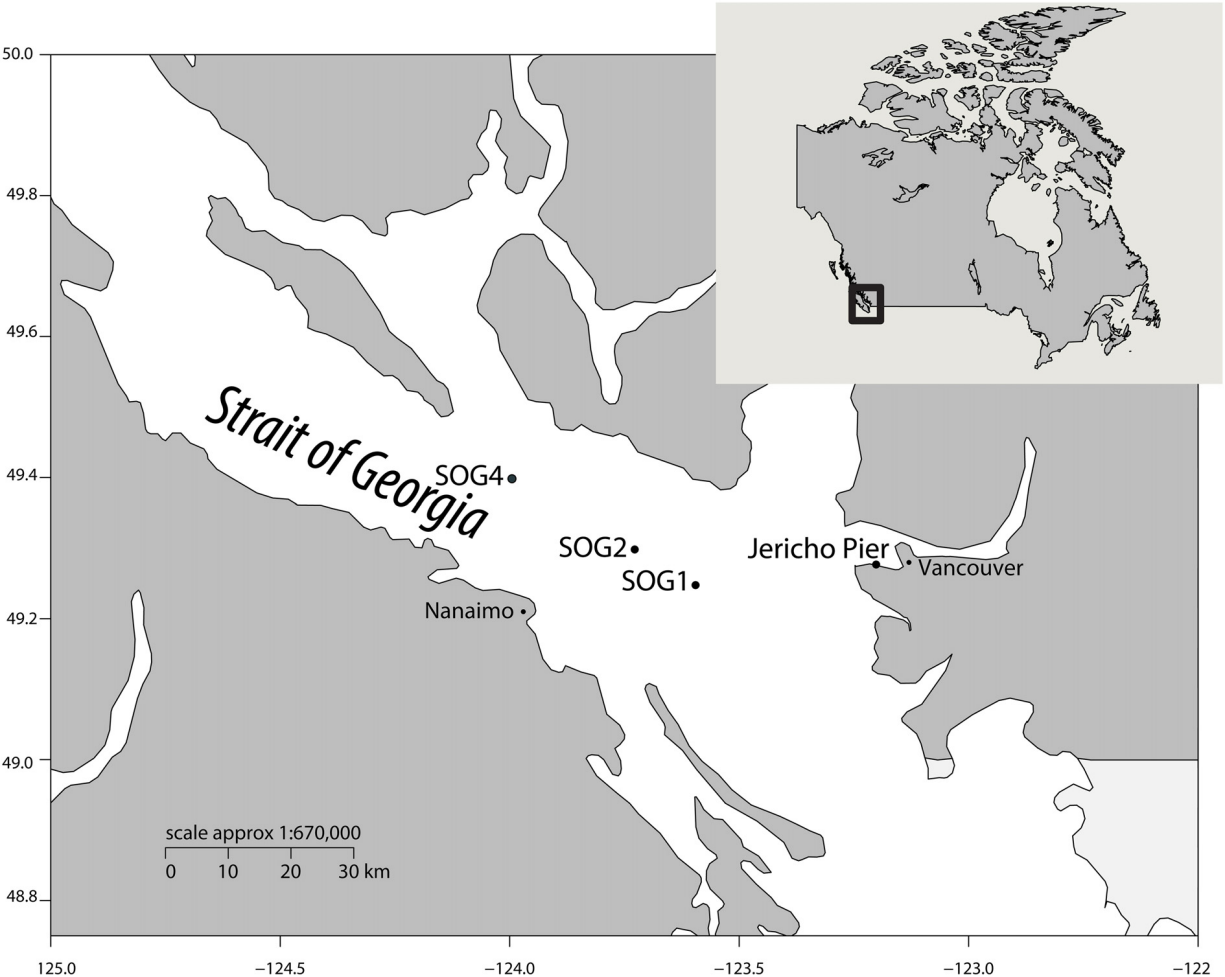
**Location of sampling sites**. Map showing the location of sampling sites within the Strait of Georgia (SOG) and Jericho Pier, adjacent to Vancouver, British Columbia, Canada. Jericho Pier was sampled in summer (JP-S) and fall (JP-F).

To assess viral communities from different coastal habitats, we collected samples from three sites in the Strait of Georgia (49°14.926N 123°35.682W, 49°17.890N 123°43.650W, and 49°23.890N 123°59.706W), and from Jericho Pier (49°16′36.73N, 123°12′05.41W) in British Columbia, Canada) (Figure 1). The Strait of Georgia (SOG) is an estuarine-influenced basin that is on average 22 km across, 222 km long and 150 m deep. The upper 50 m of SOG is where most of the variability in physical and chemical parameters occurs. Jericho Pier (JP) is adjacent to the shoreline, in a well-mixed location with mixed semidiurnal tides.

### Sample collection

On 28 July 2010, a rosette equipped with a Seabird SBE 25 CTD (equipped with Seabird SBE 43 dissolved oxygen sensor, WET Labs WETStar fluorometer, WET Labs C-Star transmissometer, Biospherical Instruments QSP2200PD PAR sensor) and General Oceanics GO-FLO bottles was used to collect 12 L of water from five depths between 2 and 16 m at each of three stations in SOG. The five depths from each station were combined to obtain three integrated water samples. The depths were selected to attempt to encompass the viral diversity at each station, and consisted of a near-surface sample, a sample from the isothermal zone below the mixed layer, and three samples spanning the chlorophyll maximum.

At JP, 60 L of water was pumped from the 1-m depth on 10 July and 12 October 2010. Salinity and temperature at JP were measured using a YSI probe (Yellow Springs, Ohio, USA). For all samples, the water was filtered through 142-mm diameter, 1.2-μm nominal pore-size glass-fiber (GC50 Advantec MFS, Dublin, CA., USA) and 0.22-μm pore-size polyvinyldine (Millipore Bedford, MA, USA) filters. The viral size fraction in the filtrate was concentrated to ~500 mL (viral concentrate) using tangential flow ultrafiltration using a 30 kDa MW prep-scale Spiral Wound TFF-6 cartridge (Millipore) (Suttle et al., 1991).

Phosphate, silicate and nitrate+nitrite concentrations were determined in duplicate 15-mL seawater samples filtered through 0.45-μm pore-size HA filters (Millipore) and stored at −20°C until air-segmented continuous-flow analysis on a AutoAnalyzer 3 (Bran & Luebbe, Norderstedt Germany). Chlorophyll *a* (Chl *a*) was determined in triplicate by filtering 100 mL of seawater onto 0.45 μm pore-size HA filters (Millipore), and storing the filters in the dark at −20°C until acetone extraction and then analyzed fluorometrically (Parsons et al., 1984). The average and standard error of the replicates was calculated for each sample.

### Nucleic-acid extraction and PCR

The viral concentrate was filtered twice through 0.22-μm pore-size Durapore PVDF filters (Millipore) in a sterile Sterivex filter unit (Millipore). The filtrate, containing virus-sized particles, was pelleted by ultracentrifugation (Beckman-Coulter, Brea, California, USA) in a SW40 rotor at 108 000 *g* for 5 h at 12°C. The pellet was resuspended overnight in 100 μL of supernatant at 4°C. To digest free DNA, the pellets were incubated with 1U/μl DNAse with a final concentration 5 mM MgCl_2_ for 3 h at room temperature. Nucleic acids were extracted using a Qiamp Viral Minelute spin kit (Qiagen, Hilden, Germany) according to the manufacturer's directions. To remove DNA, the extracted viral pellets were digested with DNase 1 (amplification grade) (Invitrogen, Carlsbad, California, USA) and the reaction was terminated by adding 2.5 mM EDTA (final concentration) and incubating for 10 min at 65°C. Complementary DNA (cDNA) was generated using Superscript III reverse transcriptase (Invitrogen) with random hexamers (50 ng/μl) as per the manufacturer. PCR was performed using primer set MPL-2 for a targeted set of the marine picornavirus-like RdRp (Culley and Steward, 2007). Each reaction mixture (final volume, 50 μl) consisted of 50 ng of cDNA, 1× (final concentration) PCR buffer (Invitrogen), 2 mM MgCl_2_, 0.2 mM of each deoxynucleoside triphosphate (Bioline, London, UK), 1 μM of each primer, and 1 U Platinum Taq DNA polymerase. The reaction was run in a PCR Express thermocycler (Hybaid, Ashford, UK) with the following conditions: 94°C for 75 s, followed by 40 cycles of denaturation at 94°C for 45 s, annealing at 43°C for 45 s, and extension at 72°C for 60 s and a final extension step of 9 min at 72°C. PCR products were cleaned using the Minelute PCR purification kit (Qiagen).

### Library prep and pyrosequencing

Libraries for each site were prepared for sequencing using NEBNext DNA Library Prep for 454 kit (New England Biolabs, Ipswich, Massachusetts, USA) following the manufacturer's directions, and using Ampure beads (Beckman-Coulter) for size selection and purification using a bead ratio of 0.8:1 beads:library. PCR amplicons were barcoded and sent for 454 Titanium pyrosequencing (Roche, Basel, Switzerland) at the Génome Québec Innovation Centre at the McGill University (Montreal, QC, Canada).

### Sequence analysis

Sequencing reads were quality trimmed using length settings between 100 and 600 bp, with a maximum of 3 primer mismatches for the specific primer, and denoised using the denoiser algorithm in QIIME (version 1.7) with default settings for Titanium data (Caporaso et al., 2010; Reeder and Knight, 2010). Sequences were checked for chimeras using UCHIME (Edgar, 2010) against a nucleotide database of RdRp sequences built using NCBI-BLAST+ by retrieving nucleotide sequences of the RdRp from *Picornavirales* viral isolates (accessed: 8 August 2012), and also using the *denovo* chimera check in UCHIME. The overlap of results from these two methods was defined as chimeric sequences, although none were found in this study. Non-chimeric sequences were queried using BLASTx (Altschul et al., 1990) with an e-value of 1e^−3^ against the database of RdRp viral isolates. All sequences with hits were retained and all sequences with no hits were then queried against the non-redundant (nr) Genbank database (Benson et al., 2007) using BLASTx with an e-value of 1e^−5^. All the sequences identified as contaminants or as unknown with only 1 read were removed. Remaining sequences were translated to amino acids using FragGeneScan with the 454_10 training option (Rho et al., 2010). Sequences were grouped into operational taxonomic units (OTUs) using UCLUST at a range of similarities from 50 to 100% (Figure S1) using the original seed sequences (centroids) as the output (Edgar, 2010). A similarity of 95% was chosen for this analysis for the following two main reasons: (1) When the NCBI conserved domain alignment for the RdRp region (all *Picornavirales)* was analyzed for percent similarity, the only sequences that displayed greater than 95% similarity in this region were strains of the same virus. (2) The sequences from the control libraries (consisting of 1 clone) clustered into 1 sequence at this percentage (see Supplemental methods). Thus clustering at 95% similarity was a way to use biological and sequence-based information to inform our choice of cut-off to collapse strain level variation and as a conservative approach to avoid variation that may be present because of the sequencing platform. Control sequences obtained by cloning and Sanger sequencing (see Supplemental Methods) were used to verify the sequence processing methodology. Raw and processed sequence data were deposited in the NCBI BioProject database ID: PRJNA267690.

### Phylogenetic analysis

All OTUs with less than 5 reads were removed and the remaining OTUs were aligned using profile alignment in Muscle (Edgar, 2004) to seed alignments of viral RdRps from the NCBI Conserved Domain Database (Marchler-Bauer et al., 2011). Sequences from other environmental surveys were clustered in the same manner as the reads in this study (using UCLUST at 95%). The clusters are cluster number followed by the Genbank accession numbers contained in that cluster. **0**: 33520549, 33520547, 33520541, 33520533, 33520527, 33520521, 33520519, 33520517, 33520515, 33520513, 33520511, 33520509; **1**: 157280772; **2**: 33520525; **3**: 157280768; **4**: 157280770; **5**: 568801536, 568801534, 568801530, 568801528, 568801510; **6**: 157280786; **7**: 157280774; **8**: 157280780; **9**: 157280788; **10**: 568801494, 568801492, 568801488, 568801482, 568801480, 568801474, 568801470, 568801466, 568801464, 568801462, 568801458; **11**: 157280776; **12**: 157280778; **13**: 568801516; **14**: 568801616, 568801614, 568801606, 568801588, 568801586, 568801584, 568801582, 568801580, 568801574, 568801572, 568801564, 568801550, 568801548, 568801540, 568801532, 568801522, 568801520, 157280784; **15**: 568801508; **16**: 568801542, 568801538, 568801518, 568801486, 568801484, 568801478; **17**: 568801612, 568801610, 568801608, 568801604, 568801602, 568801600, 568801598, 568801596, 568801594, 568801592, 568801590, 568801578, 568801570, 568801568, 568801552, 157280782; **18**: 568801576, 568801566, 568801546, 568801544; **19**: 568801562, 568801556; **20**: 568801526, 568801524, 568801514, 568801512, 568801506, 568801504, 568801502, 568801500, 568801498, 568801496, 568801490, 568801472, 568801460; **21**: 568801476, 568801468; **22**: 568801560, 568801558, 568801554; **23**: 157280744; **24**: 157280758; **25**: 157280748; **26**: 157280746; **27**: 157280742; **28**: 157280766; **29**: 157280756; **30**: 157280762, 157280754, 157280752; **31**: 157280750; **32**: 157280760; **33**: 157280764; **34**: 33520545, 33520543, 33520535, 33520531, 33520529; **35**: 33520539, 33520537, 33520523, 33520507. Alignments were masked using trimAl with the automatic heuristic (Capella-Gutierrez et al., 2009) and edited manually. ProtTest 3.2 was used for amino-acid model selection (Darriba et al., 2011) before building the initial maximum likelihood phylogenetic tree using FastTree (Price et al., 2010). Final trees were done with RA×ML using sequences belonging to viruses in the *Sequiviridae* as the outgroup, and the BLOSUM62 amino-acid model with 100 bootstraps (Stamatakis et al., 2008). The tree was visualized in R (R Core Team, 2014) using the ape package (Paradis, 2012) and edited in Figtree (http://tree.bio.ed.ac.uk/software/figtree/).

### Statistical analysis

Generation of rarefaction curves by random resampling of OTU abundances was performed using the vegan package (Oksanen et al., 2013) in R (R Core Team, 2014). Relative abundances were normalized by randomly resampling 10 000 times using vegan (Oksanen et al., 2013), normalizing to the library with the lowest number of reads and then taking the median. Rank-abundance curves were generated with ggplot2 (Wickham, 2009) using the normalized OTU relative abundances. Scripts used in this project are available as part of QIIME and custom user scripts used to process the data are available here: http://github.com/jooolia/RdRp_454_amplicons_Jericho_and_SOG; doi: 10.5281/zenodo.12509

## Results

### Environmental parameters

**Figure 2:**
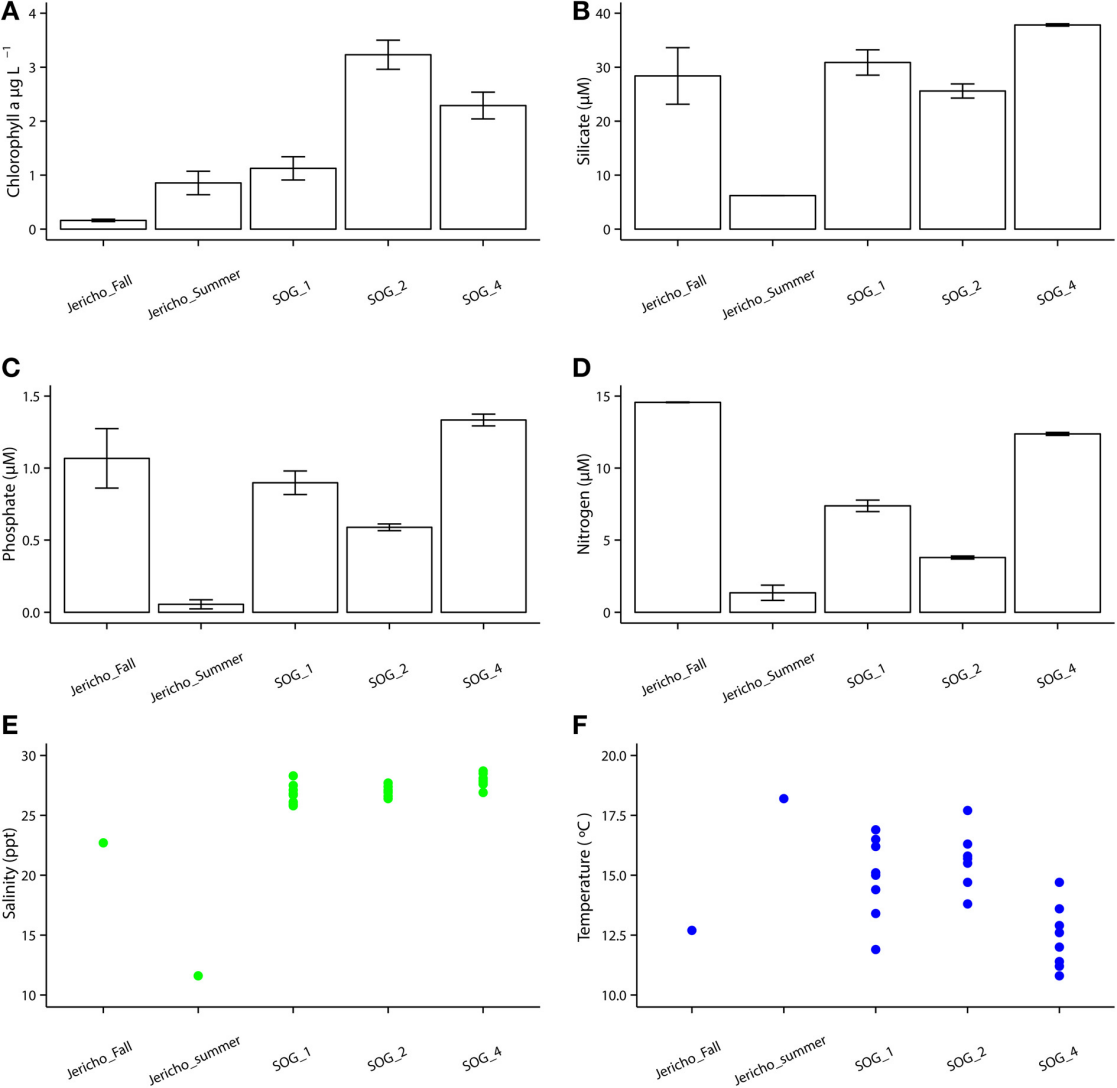
**Environmental parameters**. **(A)** Chlorophyll *a* with standard error of the mean from triplicates. **(B)** Silicate with standard error of the mean from duplicates. **(C)** Phosphate with standard error of the mean from duplicates. **(D)** Nitrate+ nitrite with standard error of the mean from duplicates. **(E)** Temperature with each point as one GO-FLO bottle (SOG samples) or from total seawater sample (Jericho samples). **(F)** Salinity with each point representing a seawater sample as one GO-FLO bottle (SOG samples) or from total seawater sample (Jericho samples).

The environmental parameters ranged widely among samples (Figure 2). Chlorophyll *a* values were lowest at Jericho Pier Fall (JP-F) at 0.16 μg L^−1^ (± 0.04) and highest at the Strait of Georgia Station 2 (SOG-2) at 3.2 μg L^−1^ (± 0.4). Silicate values for all samples were similar (range of 25.5 to 37.8 μM), except for Jericho Pier Summer (JP-S) when silicate was lower at 6.2 μM (± 0.01). Phosphate ranged between 0.90 and 1.3 μM at JP-F, SOG-1, and SOG-4, but was lower at SOG-2 (0.58 μM (± 0.03)) and lowest at JP-S (0.06 μM (± 0.04)). Nitrate + nitrite values were more variable than the other nutrients and ranged from 1.35 μM (± 0.75) at JP-S to 14.6 μM (± 0.04) at JP-F. The SOG sites were highly stratified with SOG-4 being the most stratified with a calculated mixed-layer depth of 2 m, while SOG-1 and SOG-2 were similar with a mixed-layer depth of 6 m (Table 1).

**Table 1:** **Description of samples and resulting sequencing information**.

**Location of sampling**	**Date of sample collection**	**Latitude Longitude**	Reads^*^	**Mixed layer depth (m)**
Jericho Pier Summer	10 July 2010	49°16′36.73N, 123°12′05.41W	74,096	–
Jericho Pier Fall	12 October 2010	49°16′36.73N, 123°12′05.41W	84,907	–
SOG 1	28 July 2010	49°14.926N 123°35.682W	55,197	6
SOG 2	28 July 2010	49°17.890N 123°43.650W	12,269	6
SOG 4	28 July 2010	49°23.890N 123°59.706W	73,044	2

* after quality filtering and matching to the RdRp primer set.

### Analysis of RdRp sequences

**Figure 3:**
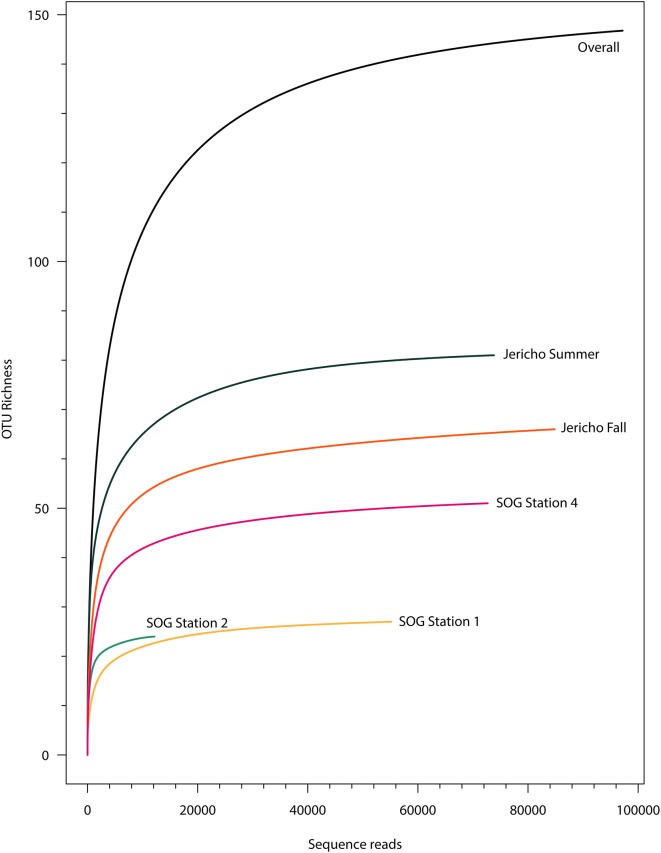
**Rarefaction curves**. Rarefaction analysis of RdRp amplicons based on Chao1 richness analysis of operational taxonomic units (OTUs) at 95% similarity. Rarefaction curves were resampled using number of reads recovered per library. Rarefaction curves plateau indicating adequate sequencing for these samples.

After quality filtering to remove homopolymers and contaminating reads, 300 180 reads were recovered from the 5 libraries of RdRp amplicons. At all sites the rarefaction curves plateaued indicating that the depth of sampling was adequate to assess the communities (Figure 3). From these reads, 265 unique OTUs (at 95% similarity) were identified, including 108 singletons. For further analysis OTUs were excluded that did not contain recognizable RdRp motifs (Koonin, 1991; Le Gall et al., 2008), generally did not align well with other RdRp sequences and those that were not present in any sample after normalization.

**Figure 4:**
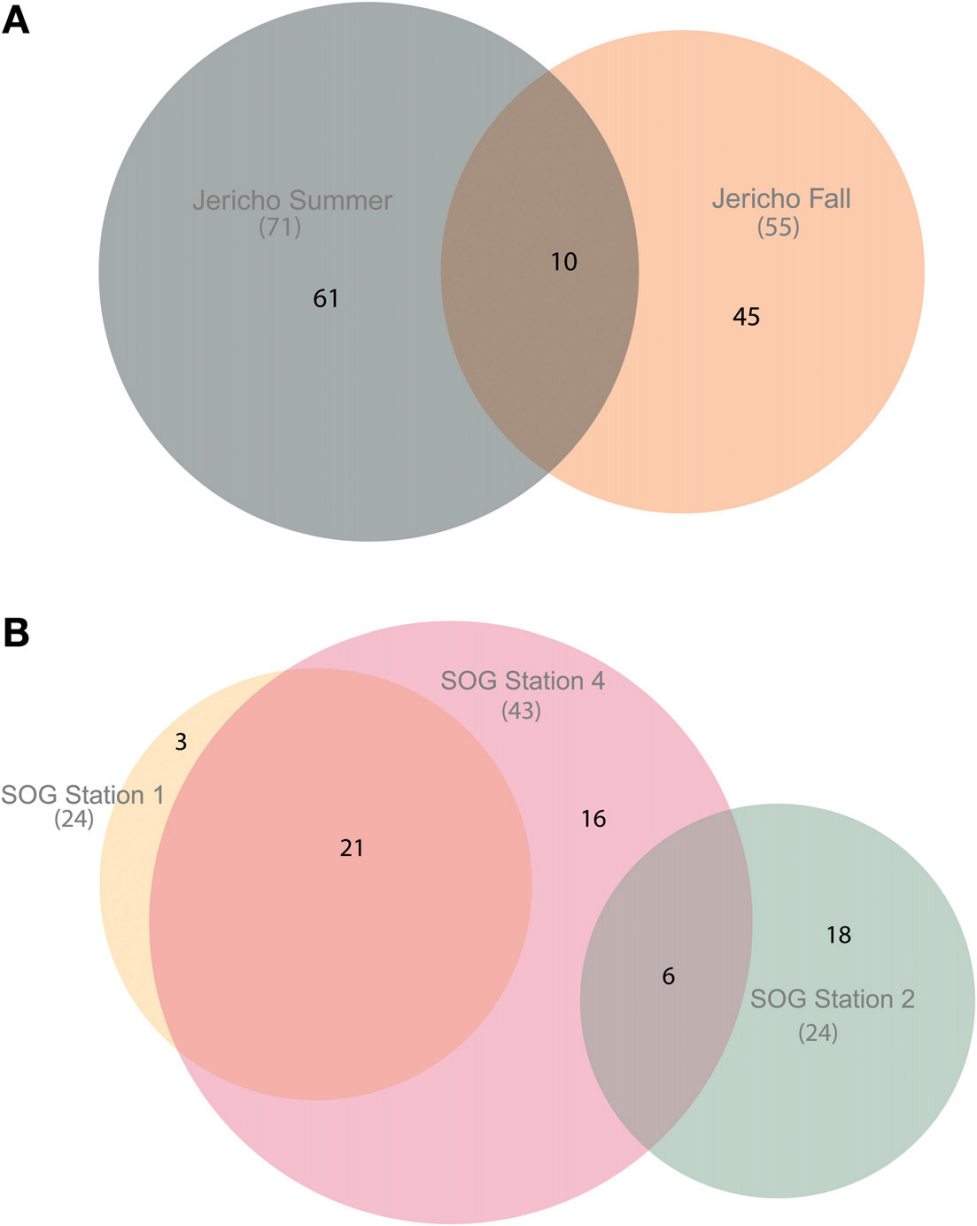
**Euler diagrams of normalized RdRp OTUs**. **(A)** Euler diagram of Jericho Pier samples. **(B)** Euler diagram of SOG samples. The OTUs presented were from reads clustered at 95% similarity, comprise only OTUs that could be aligned to the NCBI CDD RdRp alignment, and contained the RdRp motif C. The diagrams were constructed using the venneuler() algorithm (Wilkinson, 2011). The size of the circles is approximately proportional to the number of OTUs recovered per site. The overlap in the diagram describes OTUs that were found at multiple sites and the non-overlapping areas describe OTUs that were unique to that site.

Using the above criteria there were 145 OTUs identified in all samples, and between 24 to 71 OTUs per site. The Jericho Pier samples had 116 OTUs of which only 10 (8.6%) were shared between sampling times (Figure 4). JP-S had the highest richness with 71 OTUs, of which 59 (83 %) were unique, while JP-F had the second highest richness (49 OTUs), of which 45 (92 %) were unique. The SOG sites together had 64 OTUs, none of which were shared among all sites. SOG-1 and SOG-2 had the lowest number of OTUs (24). SOG-1 had only three OTUs which were unique. However, 21 (33%) were shared between SOG-1 and SOG-4, and 6 (9%) between SOG-2 and SOG-4. The majority of OTUs (75%) from SOG-2 were unique, whereas most OTUs from the other SOG sites were shared with other sites (87% for SOG-1 and 63% for SOG-4).

**Figure 5:**
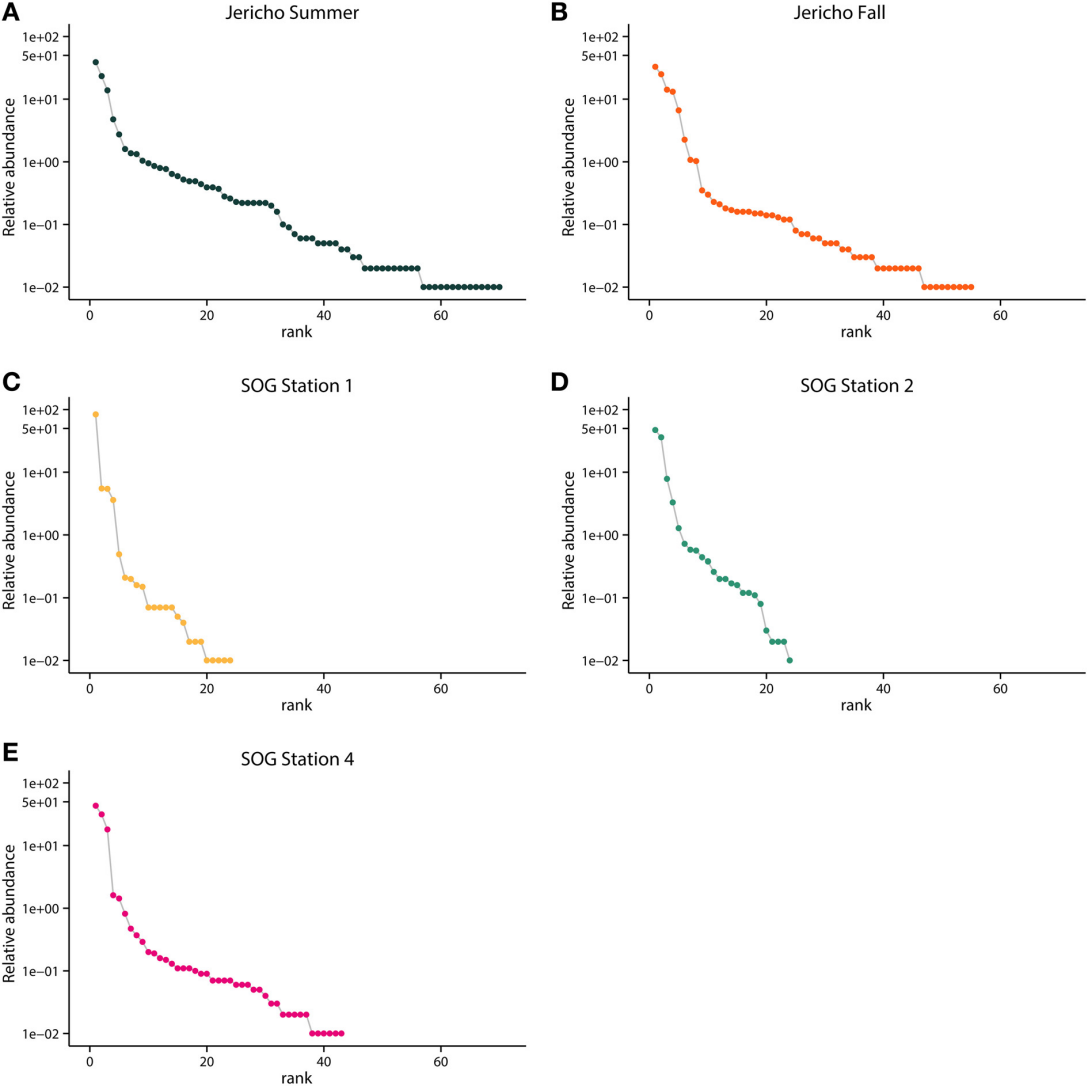
**Rank abundance by site**. Relative abundance of OTUs in each sample ordered by rank abundance: **(A)** Jericho Summer, **(B)** Jericho Fall, **(C)** SOG Station 1, **(D)** SOG Station 2, **(E)** SOG Station 4. OTUs were clustered at 95% amino acid similarity and OTU relative abundances were normalized to the sample with the lowest number of reads.

Rank abundance curves of the viral OTUs showed that at each site most sequences were assigned to only a few OTUs (Figure 5). JP-S had the highest richness but the shallowest slope of these curves, demonstrating more evenness in the abundance of OTUs than at the other sites. SOG-4 and JP-S had similar rank abundance curves that were much shallower that those of SOG-1 and SOG-2 (Figure 5).

**Figure 6:**
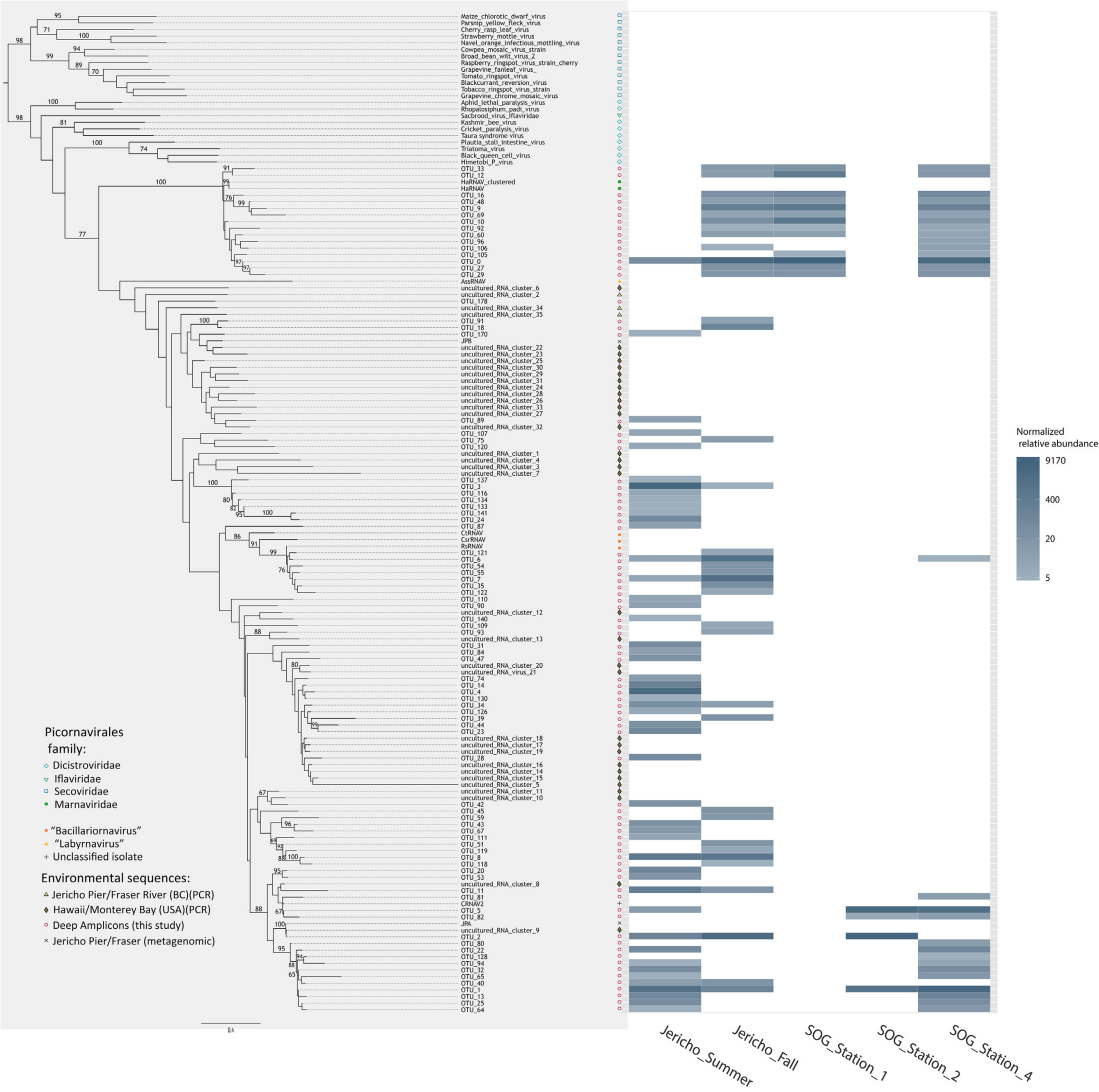
**Phylogenetic tree with heatmap**. Maximum likelihood (RA×ML) tree of RdRp OTUs that contained more than 5 reads and relevant *Picornavirales* sequences. Bootstrap values above 65% are labeled. Adjacent to the tips of the tree is a heatmap displaying the relative abundance of each OTU at 95% similarity by site. OTU relative abundances were normalized to the sample with the lowest number of reads.

The OTUs that were observed in more than 5 reads were placed in phylogenetic context using a maximum likelihood RA×ML tree (Stamatakis et al., 2008) with sequences from previous RdRp gene surveys and isolated viruses (Figure 6). OTUs from this study fell within a well-supported clade that includes all the marine isolates belonging to the *Picornavirales*. Within this group there was a well-supported divide between OTUs grouping in the *Marnaviridae* clade and those grouping with sequences from viruses infecting diatoms and a thraustochytrid. The overall tree topology is not well supported, although there are a number of well-supported clades containing OTUs from this study and other environmental sequences.

The *Marnaviridae* clade had the greatest number of OTUs (10) associated with it; whereas, very few OTUs (only OTUs 89, 107, 75, and 120) from this study were assigned to clades primarily from Hawaii (Culley and Steward, 2007; Culley et al., 2014). No clade contained OTUs from all sites. The Jericho Pier samples were the most phylogenetically diverse (Figure 6, Table 2), and contained OTUs (e.g., OTUs 6, 7, 35, 31, 84, 47, 14, 4, 34, 39, 44, 23, 8, 20) that fell into clades that did not contain OTUs from any of the SOG samples. Some clades contained OTUs from both JP-S and JP-F samples; however, many OTUs within the clades were unique to one Jericho Pier sample. Phylogenetic diversity differed among samples, except for OTUs from the two SOG sites with deeper mixed layers, some of which were present in different clades resulting in similar phylogenetic diversity (Table 2).

**Table 2:** **Phylogenetic diversity, species richness**.

	**Phylogenetic diversity**	**Species Richness**
Jericho Pier Summer	16.25	46.00
Jericho Pier Fall	13.62	30.00
SOG Station 1	3.89	9.00
SOG Station 2	3.26	4.00
SOG Station 4	8.35	24.00

Phylogenetic diversity (PD) is calculated as in Faith (1992). OTUs were the same as used in the construction of the phylogenetic tree and must have included 5 or more reads.

The Strait of Georgia (SOG) sites were sampled within hours of each other, and the water at each site was pooled from multiple depths above, below, and across the chlorophyll maximum. One of the most striking differences among sites was that SOG-1 and SOG -2 had mixed layer depths of 6 m; whereas SOG-4 had a mixed-layer depth of 2 m, and much higher richness and phylogenetic diversity. Sites SOG-1 and SOG-2 had the lowest phylogenetic diversity (Table 2). All the OTUs found at SOG-1 (33, 12, 16, 9, 10, 27, 29) were within the *Marnaviridae* clade; similarly, all OTUs (5, 82, 2, 1) from SOG-2 were within one distantly related clade. In both cases OTUs from these clades occurred at SOG-4. SOG-2 did not have the high numbers of HaRNAV-related viruses that were found in all other samples.

## Discussion

Pyrosequencing of RdRp gene fragments from coastal samples uncovered much greater genetic diversity than in previous gene surveys (Culley et al., 2003, 2014; Culley and Steward, 2007) and revealed many previously unknown taxonomic groups within the *Picornavirales*. As well, striking differences in the taxonomic richness among samples implies that these viruses infect a wide variety of eukaryotic plankton, but that the mortality imposed on some taxa is highly variable across space and time. Other taxonomic groups within the *Picornavirales* were more widespread, suggesting that infection of some planktonic taxa is more widespread and persistent. These results and their implications are discussed in detail below.

### Expanding the known diversity of *Picornavirales*

The high depth of sequencing and limited diversity in each library (Figure 3) gives high confidence that the population structure of RdRp amplicons in each sample has been well characterized (Kemp and Aller, 2004). Although some sequences were closely related to those found in previous studies (Culley et al., 2003; Culley and Steward, 2007) (Figure 6), many OTUs formed new clades. Many OTUs were related to *Heterosigma akashiwo* RNA virus (HaRNAV) that infects the toxic bloom-forming raphidophyte *Heterosigma akashiwo* (Tai et al., 2003). HaRNAV is the type virus of the family *Marnaviridae* (Lang et al., 2004); it has a genome of about 9.1 kb and a high burst size as indicated by the large crystalline arrays of particles in the cytoplasm of infected cells (Tai et al., 2003). HaRNAV was isolated from coastal waters in British Columbia, Canada (Tai et al., 2003) and can remain infectious for many years in sediments (Lawrence and Suttle, 2004). Interestingly, HaRNAV was isolated from the same area as the present study, and appeared ancestral to many of the recovered sequences based on the phylogeny. For example, OTU 0 was most abundant (18,034 reads after rarefaction) and clustered closely with HaRNAV, although the sequence was only 76.4% similar at the amino-acid level. However, other OTUs in the cluster ranged between 55 and 79% similar to HaRNAV, which is low compared to amino-acid similarities of other RNA viruses within a family that usually have greater than 90% aa similarity (Ng et al., 2012).

### Distinct communities occurred in different seasons at the same location

While only 8.6% of the OTUs from Jericho Pier were shared between dates, both samples had similar evenness, although the summer sample had greater richness (Figure 5, Table 2). The small overlap in OTUs between sampling dates is not surprising given the very different conditions between July and October (Figure 1), and the dynamic nature of planktonic communities in response to environmental changes. At the same location, dsDNA viruses belonging to the *Phycodnaviridae*, which infect eukaryotic phytoplankton, varied seasonally based on fingerprint analyses of DNA polymerase gene fragments using denaturing gradient gel electrophoresis; however, some OTUs persisted for extended periods (Short and Suttle, 2003). Similarly, the composition of other aquatic viral communities has been shown to be dynamic although some OTUs persist (Djikeng et al., 2009; Rodriguez-Brito et al., 2010), and in some cases have repeatable seasonal patterns (Chow and Fuhrman, 2012; Clasen et al., 2013; Marston et al., 2013). With only single samples from summer and fall, inferences about dynamics cannot be made from our data.

One of the few taxonomic groups that occurred in both the summer (JP-S) and fall (JP-F) samples from Jericho Pier was related to HaRNAV (Figure 6). There was greater diversity of OTUs in this clade in JP-F, even though JP-S had higher richness and higher phylogenetic diversity overall. This is unlike bacterial and phytoplankton communities that tend to be more diverse in winter (Zingone et al., 2009; Ladau et al., 2013). However, the RdRp primers target a specific subset of the viral community that might not reflect the overall taxonomic diversity.

Based on genome organization and sequence identity, RNA viruses that infect diatoms have been assigned to the genus *Bacillarnavirus*, that includes *Rhizosolenia setigera* RNA virus (RsRNAV) (Nagasaki et al., 2004)*, Chaetoceros tenuissimus* Meunier RNA virus (CtenRNAV) (Shirai et al., 2008) and *Chaetoceros socialis* f. radians RNA virus (CsfrRNAV) (Tomaru et al., 2009). In the JP-F sample, the most relatively abundant cluster grouped with RsRNAV that infects the marine diatom *Rhizosolenia setigera* (Nagasaki et al., 2004). This corresponded with the highest levels of nitrate + nitrite, which is often associated with high diatom abundances (Zingone et al., 2009); hence, these OTUs are likely associated with viruses infecting diatoms.

### Distinct communities occurred at geographically proximate sites

Areas of higher habitat diversity, such as stratified water layers, generally have higher biological richness (Klopfer and MacArthur, 1960; Chesson, 2000), and this is consistent with the much higher richness and phylogenetic diversity found at SOG-4, which was the most stratified site and included the most abundant OTUs from SOG-1 and SOG-2. Most OTUs from SOG-1 clustered in the *Marnaviridae* clade, while most SOG-2 OTUs clustered in a phylogenetically distant clade. Given that we have used very conservative clustering, and that dsDNA viruses infecting phytoplankton are strain specific and have phylogenies that are congruent with their hosts (Clasen and Suttle, 2009; Bellec et al., 2014), and that RNA viruses infecting diatoms and the dinoflagellate, *Heterocapsa circularisquama* (Nagasaki et al., 2005) are host-specific, it implies that closely related OTUs infect closely related taxa of phytoplankton. Hence, it suggests that the most abundant viruses at these three locations infect different species.

There are few clear patterns in the spatial distribution of viruses in marine waters where geographically distant sites are connected by currents and mixing. The best examples are for cyanophages. For instance, when looking at local variation in cyanophages isolated at sites in Southern New England, 72% of the viral OTUs were shared between at least 2 sites (Marston et al., 2013); however, between Bermuda and Southern New England only 2 OTUs overlapped and they comprised only 0.6% of the isolates. Yet, clear patterns of cyanophage OTU distribution by depth occurred in areas adjacent to the SOG when assessed using community fingerprinting (Frederickson et al., 2003). The biggest differences with depth occurred in stratified water in which some OTUs were present at all depths, while others were only present at specific depths, even though the samples were collected only meters apart (Frederickson et al., 2003). These viruses infect cyanobacteria, as opposed to the picorna-like viruses, which likely infect protistan plankton. Nonetheless, the factors governing the distribution of cyanobacterial and protistan hosts are likely similar; hence, different OTUs would be expected to occur in environments with different vertical structure (stratification) of the water column.

Rank abundance curves showed that SOG-1 and SOG-2 were the least even communities (Figure 5). Overall, at most sites four to five viral OTUs were most abundant (Figure 5) similar to other reports for aquatic viral communities in which a few viruses dominate, but most of the diversity comes from rarer viruses (Angly et al., 2006; Suttle, 2007). Our targeted approach showed that the picornavirus-like virus communities at SOG and JP were dominated by only a few genotypes, supporting previous metagenomic results showing that the OTU distributions of RNA viruses in SOG and JP were highly uneven with little overlap between sites (Culley et al., 2006).

### Each OTU likely represents a single lytic event

Given that the hosts of marine *Picornavirales* isolates are protists, and that protists are the most abundant eukaryotes in the sea, it is likely that the majority of OTUs recovered in this study are from viruses that infect these unicellular marine eukaryotes. These eukaryotic communities are highly dynamic and change throughout the year based on environmental and biological factors (Larsen et al., 2004). Since viral infection is usually host specific, the diversity in marine viral communities is a reflection of the underlying diversity of the marine eukaryotic hosts. Moreover, viral propagation is dependent on host encounter rates and is proportional to host-cell abundance (Murray and Jackson, 1992); hence the most abundant taxa will be most likely to encounter and propagate a viral infection, giving the opportunity for rarer species to increase in abundance and promoting diversity (Thingstad, 2000; Winter et al., 2010). Since our study was not over time it is difficult to evaluate whether these data support the Bank model (Breitbart and Rohwer, 2005), however, some taxa were found at one site, but not a similar nearby site, thus these taxa could be present at background levels at some sites and more abundant in others.

It is probable that the most abundant OTUs in these data are from recent lysis of host taxa. An error rate for replication of RNA viruses of about 1 bp mutation per generation (9000 bp genome × 0.0001 error rate per base pair = 1 bp; Holmes, 2009), and a lower-end burst-size estimate of 1000 particles for marine viruses in the *Picornavirales* that infect protists (Lang et al., 2009), would produce about 1000 different genomes from each lysed cell. For the amplified 500 bp RdRp gene fragment there is a 0.00056% chance of an error in 1 generation, assuming that mutations are distributed evenly in the genome (Sanjuan et al., 2010; Combe and Sanjuán, 2014). Consequently, even with the relatively high error rates of RNA replication, when grouped at 95% similarity at the amino acid level, all of the sequences from a lytic event should fall within a single OTU. The half-life for decay of viral infectivity and particles in the surface mixed layer is typically a few hours (Heldal and Bratbak, 1991; Suttle and Chen, 1992; Noble and Fuhrman, 1997; Bettarel et al., 2009); thus the recovered viral OTUs were likely from recent lytic events. Furthermore, considering the specificity of viruses infecting protists (Short, 2012), each OTU probably stems from viruses infecting a single host taxon. Thus, these data imply that infection of marine protists by viruses in the *Picornavirales* is not only pervasive, but likely involves a wide diversity of host taxa; hence, these viruses are likely important structuring elements for phytoplankton communities that influence nutrient cycling and energy flow.

### Amplicon deep sequencing as an approach for estimating viral diversity

Amplicon deep sequencing is a sensitive and high-resolution approach for examining microbial community dynamics over time and space (Caporaso et al., 2011; Gobet et al., 2012; Gibbons et al., 2013). Careful quality trimming of sequences and removal of singletons is essential for reliable results (Zhou et al., 2011) since errors in sequences will inflate estimates of diversity. With careful data processing and analysis, amplicon deep sequencing is as accurate for assessing community composition and diversity as cloning and Sanger sequencing (Amend et al., 2010), but with much greater depth of coverage of the community.

There are potential biases associated with reverse transcription with random hexamers (which can decrease yield and could inflate diversity) (Zhang and Byrne, 1999), template amplification by PCR (Lee et al., 2012) and with using highly degenerate primers that target a specific part of the community containing many different templates (Culley and Steward, 2007). A danger of the high cycle number can be diversity overestimates which can come from the increasing number of chimeric sequences produced with greater cycle number (Qiu et al., 2001). The sequences were processed with caution considering the high number of PCR cycles employed in this study. Chimera checking *denovo* was used to look for chimeric sequences originating from two higher abundance reads, and reference-based chimera checking was used a database of RdRps from isolated viruses to correct for this potential error. In addition, a conservative cut-off was used of only OTUs comprising more than 5 reads that aligned to the conserved domain alignment.

Read abundance of OTUs can be considered semi-quantitative and good for comparisons of richness and diversity among samples (but not for absolute counts of genes) (Amend et al., 2010; Pinto and Raskin, 2012; Ibarbalz et al., 2014). Moreover, by using control sequences obtained by cloning and Sanger sequencing alongside pyrosequenced libraries containing the same sequence (Supplemental Methods, Figure S2) we verified that amplicon deep sequencing and our sequence processing methodology recovered accurate environmental viral sequences and non-inflated estimates of richness like in studies for bacterial amplicons (Sogin et al., 2006; Huse et al., 2008; Kirchman et al., 2010; Caporaso et al., 2011) and clinical viral studies (Romano et al., 2013; Watson et al., 2013).

## Conclusion

Amplicon deep sequencing of RdRp gene fragments using 454 pyrosequencing revealed the richness and population structure of marine *Picornavirales* in five coastal samples. The known diversity of viruses in this group was greatly increased with 145 OTUs that differed by at least 5% at the amino-acid level. There were between 24 and 71 OTUs in each sample, with distinct patterns of OTU distribution, richness and diversity among samples. There was little overlap between viral OTUs collected at the same site in summer and fall, and among samples collected 20 km apart on the same day. The high temporal and spatial diversity in RdRp sequences is consistent with viral communities that turnover rapidly, and episodic infection of a wide diversity of protistan hosts. The low overlap in OTUs and phylogenetic diversity among samples implies a dynamic landscape of viral infection and supports the idea that marine picorna-like viruses are important pathogens of marine protists that have an important role in structuring marine planktonic communities, and in nutrient cycling and energy transfer among trophic levels. Ultimately, further study is needed to disentangle the temporal and spatial drivers of these communities.

### Conflict of interest statement

The authors declare that the research was conducted in the absence of any commercial or financial relationships that could be construed as a potential conflict of interest.
