# Supplementary material for: High temporal and spatial diversity in marine RNA viruses implies that they have an important role in mortality and structuring plankton communities
Source: Front Microbiol. 2014 Dec 15;5:703. doi: 10.3389/fmicb.2014.00703 (PMC4266044; doi:10.3389/fmicb.2014.00703)
Supplement: Supplementary file 1 [file DataSheet1.DOCX]

***Supplementary Material***

**High temporal and spatial diversity in marine RNA viruses (Picornavirales) revealed by high-throughput sequencing implies an important role in mortality and structure of plankton communities**

**Julia A Gustavsen^1^, Danielle M Winget^2^, Xi Tian^3^ and Curtis A Suttle^1, 4 *^**

^1^Department of Earth, Ocean and Atmospheric Sciences, University of British Columbia, Vancouver, B.C., Canada

^2^ Seattle Pacific University, Seattle, WA., U.S.A.

^3^Bioinformatics Graduate Program, Faculty of Science, University of British Columbia, Vancouver, B.C., Canada

^4^Departments of Botany, and Microbiology & Immunology, University of British Columbia, Vancouver, B.C., Canada, and Canadian Institute for Advanced Research, Toronto, ON, Canada

*** Correspondence:**

Dr. Curtis Suttle

Department of Earth, Ocean & Atmospheric Sciences,

The University of British Columbia,

2020 - 2207 Main Mall,

Vancouver, BC, V6T 1Z4, Canada

[suttle@science.ubc.ca](mailto:suttle@science.ubc.ca)

1. **Supplementary Data**

Control libraries:

Methods:

Two control libraries were prepared in addition to the 5 sample libraries prepared. To get the Sanger sequence for 1 amplicon, from one PCR amplification, a single sequence was cloned into TOPO TA vector (Invitrogen) and transformed into E. coli grown in LB + amp at 37°C overnight. Colony PCR was performed on several clones. Positive PCR products were cleaned using the Qiagen Minelute PCR cleanup and sequenced using the M13 forward primer at NAPS (UBC) on an ABI sequencer using Big Dye Chemistry. To make the unamplified control sequence the plasmid containing the clone was grown in large quantities overnight (6x75ml cultures). Cells were harvested by centrifugation at 3200 g at 4°C for 20 min. The plasmids were extracted using the Qiagen mini-prep plasmid kit and digested with Ecor I for 2 h (600 µl DNA, 60 µl React 3 buffer, 300 µl Ecor I, 38.5 µl H20) to cut out the product from the vector. The digested extracts were run on 1.5% agarose gels and the Qiagen Minelute gel extraction kit was to purify the desired cut product. The purified cut product was processed in library preparation like the other samples (see Materials and Methods). To make the amplified control sequences, the purified digested material was used as template in a PCR reaction as detailed above and the product was used in library preparation.

Results:

The control libraries contained a total of 94 reads. Three reads were recovered from the non-amplified control sequence and 91 reads from the amplified cloned sequence. The non-amplified control sequences had no errors. However, there were only 3 reads recovered from that library. Therefore, it is difficult to compare to the amplified library. The 91 reads from the amplified library contained some sequences with insertions and some with erroneous base-calls (Figure S 2).

These control libraries enabled confident testing of the error-correction algorithm(Reeder and Knight, 2010). There were errors such as homopolymers and insertions attributable to PCR amplification and 454 pyrosequencing. However, the denoiser algorithm adequately corrected the viral OTU reads.

1. **Supplementary Figures and Tables**

## Suplementary Figures


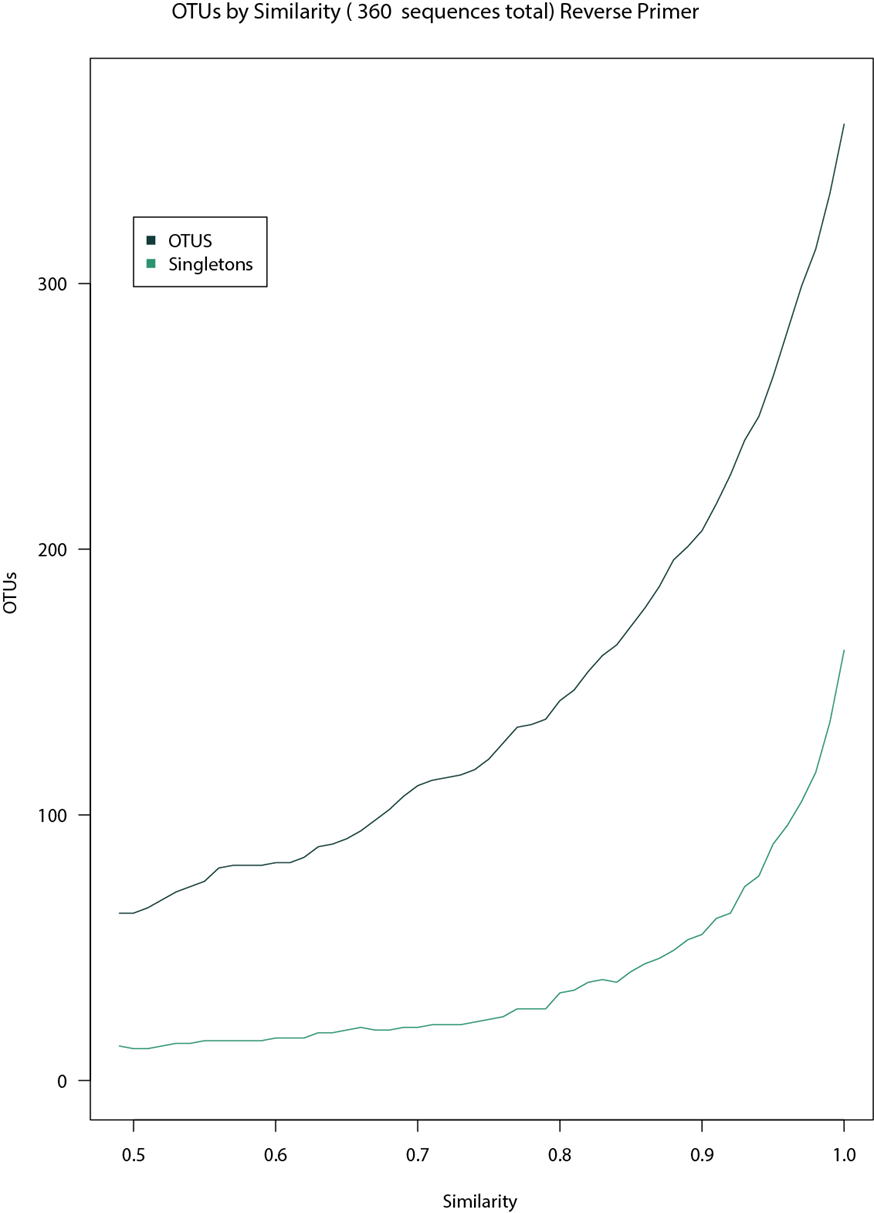


Supplementary Figure 1. Percent similarity vs. number of OTUs:All sequences were translated to amino acids using FragGeneScan with the 454_10 training option (Rho et al., 2010) and were clustered with uclust (Edgar, 2010).


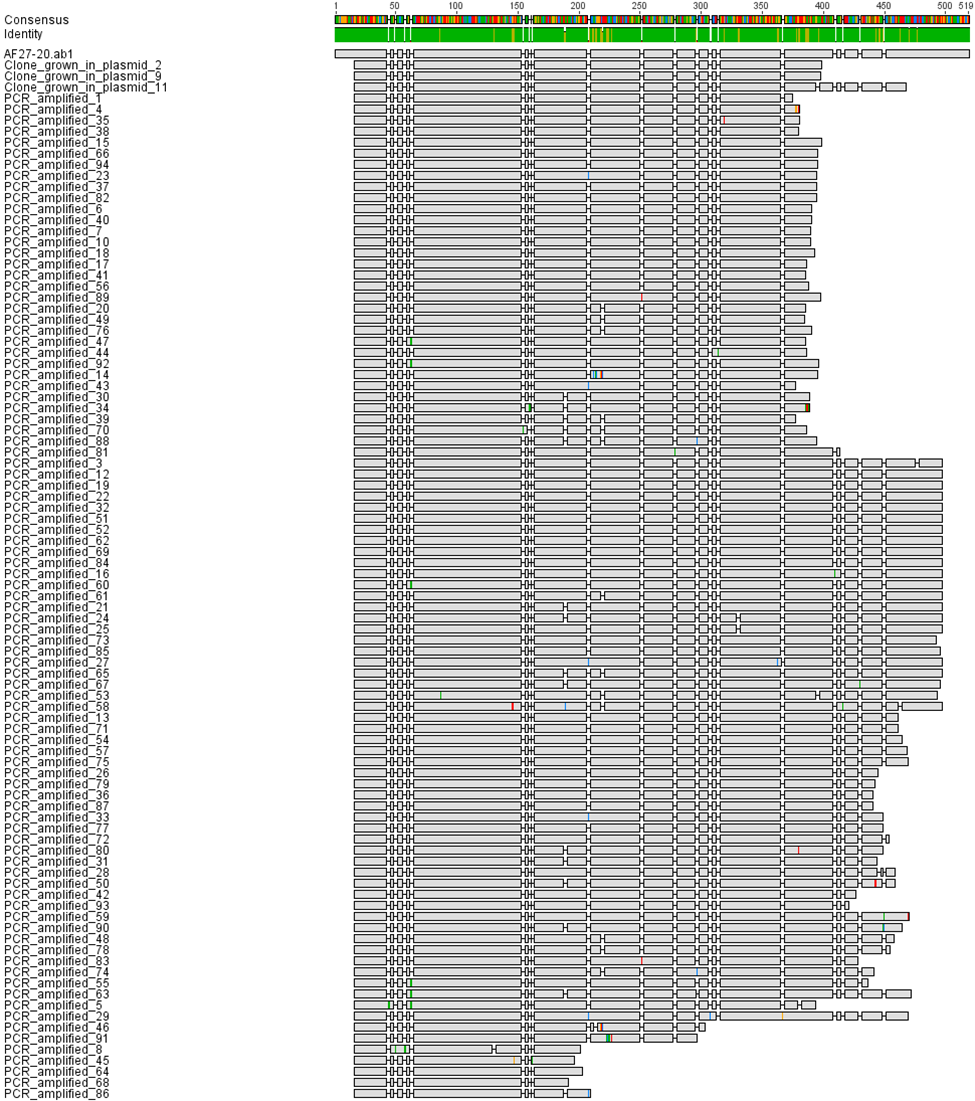


Supplementary Figure 2. Control sequence reads clustered at 95% similarity:All sequences were translated to amino acids using FragGeneScan with the 454_10 training option (Rho et al., 2010) and clustered with uclust at 95% similarity using centroids as the output (Edgar, 2010). Sequences were aligned using default parameters for MUSCLE(Edgar, 2004). Mismatches in clustered sequences are highlighted in colours. Screenshot was taken from Geneious ((6.1.6) created by Biomatters. <http://www.geneious.com/>).


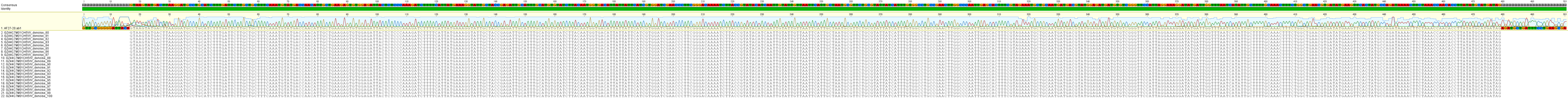


Supplementary Figure 3. Control sequence and PCR amplified reads denoised at different percentages using the QIIME denoiser Titanium settings (Reeder and Knight, 2010). All sequences were translated to amino acids using FragGeneScan with the 454_10 training option (Rho et al., 2010) and clustered with uclust at 95% similarity using centroids as the output (Edgar, 2010). Sequences were aligned using default parameters for MUSCLE (Edgar, 2004). Screenshot was taken from Geneious (v.6.1.6 created by Biomatters. http://www.geneious.com/).

1. **References**

Edgar, R. C. (2004). MUSCLE: multiple sequence alignment with high accuracy and high throughput. *Nucleic Acids Res.* 32, 1792–1797. doi:10.1093/nar/gkh340.

Edgar, R. C. (2010). Search and clustering orders of magnitude faster than BLAST. *Bioinformatics* 26, 2460–2461. doi:10.1093/bioinformatics/btq461.

Reeder, J., and Knight, R. (2010). Rapidly denoising pyrosequencing amplicon reads by exploiting rank-abundance distributions. *Nat. Methods* 7, 668–669. doi:10.1038/nmeth0910-668b.

Rho, M., Tang, H., and Ye, Y. (2010). FragGeneScan: predicting genes in short and error-prone reads. *Nucleic Acids Res.* 38, e191–e191. doi:10.1093/nar/gkq747.
